# Modification of the Mechanical Properties of Photosensitive Resin by Using Biobased Fillers During Stereolithography (SLA) 3D Printing

**DOI:** 10.3390/ma18122699

**Published:** 2025-06-08

**Authors:** Miroslav Müller, Jiří Urban, Jaroslava Svobodová, Rajesh Kumar Mishra

**Affiliations:** Department of Material Science and Manufacturing Technology, Faculty of Engineering, Czech University of Life Sciences Prague, Kamycka 129, Suchdol, 165 00 Prague, Czech Republic; muller@tf.czu.cz (M.M.); urbanjiri@tf.czu.cz (J.U.); svobodovajaroslava@tf.czu.cz (J.S.)

**Keywords:** 3D printing, additive manufacturing technologies, biological fillers, composite, mechanical properties, stereolithography (SLA)

## Abstract

This paper is focused on the modification of commercial resin by using biobased fillers during stereolithography (SLA) 3D printing. This research aims to create a composite material with a matrix made of commercially available photosensitive resin modified with a filler based on secondary raw materials and materials formed as by-products in the processing of biological materials. The research determines the effect of different fillers on the tensile properties and hardness of samples printed using SLA 3D printing, and it also investigates their integrity using SEM analysis. This study aims to evaluate the feasibility of using these fillers for producing 3D-printed parts with SLA technology. The results of this study open up new possibilities for designing modified composite materials based on additive SLA 3D-printing technology using biological fillers. Within the framework of research activities, a positive effect on tensile properties and an improved interfacial interface between the matrix and the filler was demonstrated for several tested fillers. Significant increases in tensile strength of up to 22% occurred in composite systems filled with cotton flakes (CF), miscanthus (MS), walnut (WN), spruce tree (SB), wheat (WT) and eggshells (ES). Significant potential for further research activities and added value was shown by most of the tested bio-fillers. A significant contribution of the current research is the demonstration of the improved mechanical performance of photosensitive resin modified with natural fillers.

## 1. Introduction

Composites based on polymeric materials have been of considerable interest to researchers for quite a long time due to their enhanced mechanical properties and the possibility of utilising secondary raw materials and increasing the proportion of biological materials used [1,2,3,4]. Nowadays, increased attention is being paid to the use of fillers in polymeric materials in the field of additive technology [5,6,7]. A significant number of additive technologies use polymeric materials [3,8,9].

Additive manufacturing 3D-printing technologies, which were originally seen as a rapid prototyping tool, are now occupying a significant proportion of conventional manufacturing [10]. The use of additive technologies in polymer processing has been widely adopted in several industries, mainly due to the acceleration of development and the ability for this development to be quickly reflected in production changes, leading to time and resource savings [9].

The two dominant groups in the field of additive technologies using polymer materials are fused deposition modelling (FDM) and stereolithographic printing (SLA), see refs. [3,9]. FDM printing uses thermoplastics while SLA uses reactive plastics—more specifically photopolymers [3,11,12,13]. SLA technology is designed for high-quality, complex and high-precision products [14]. Current research activities, mainly focusing on FDM or 3D-printing methods, are relatively well documented. These include studies on printing parameters, various fillers used as additives, degradation processes and investigations into the durability of printed materials, particularly regarding fatigue testing [3,11,12,13].

However, one of the main trends in additive technologies is photosensitive resins (otherwise known as SLA resins), and research in this area is focused mainly on the addition of both synthetic and biological fillers (nanoclay, carbon, ceramics, carbon black, carbon nanofibre, nanocellulose, carbon nanotube, wood flour, etc.) [7,10,15,16]. Three-dimensional printing with (SLA) technology uses ultraviolet (UV) light to cure liquid photosensitive resins, which belong to the group of reactive plastics [9,17,18,19]. The fundamental chemical mechanism utilised in curing photosensitive resins in 3D printing is radical photopolymerisation by the absorption of ultraviolet light from photoinitiators, which ensures the curing process [17,18,20]. Based on the state the photosensitive resin is in before printing, experimentation is possible in the field of reinforcement additions [1,21]. However, higher filler concentrations cause significant stability, sedimentation and inhomogeneity problems [3,21,22,23].

It has been reported that SLA achieves better mechanical properties than FDM. Hence, it has been more widely adopted in recent years [19]. Nevertheless, most of the literature points to a shortcoming of this technology; i.e., SLA 3D printing has a significant drawback in that it usually results in products with lower mechanical properties compared to those produced with conventional additive methods [7]. Very often, brittle fracture occurs in photosensitive resins or in parts printed by SLA printing [24]. This implies that the mechanical performance of various components printed using SLA technology often does not meet strict criteria required for industrial applications due to lower tensile strength values [24,25]. This limitation of SLA technology has led to intensive research and necessitates the development of new strategies in order to improve the mechanical properties of SLA-based 3D-printed parts [25]. One strategy is to add filler. Several studies have already demonstrated the positive effect of filler addition in SLA printing. Such fillers have included cotton flakes, wood flour, carbon fibre, rabbit hair, SiO_2_, Al microparticles, waste from cereal production, etc. [1,7,16,24,25,26,27,28,29]. There are several types of filler materials that can reinforce photosensitive resins [7]. The important fact is that adding fillers to polymeric liquid photosensitive resins affects the dimensional stability and accuracy, the uniformity of the surface, and the strength of the component as well as the overall complexity of the process [10].

The basic objective of current research activities is not only to assess how mechanical properties are impacted by the addition of filler but especially to increase the environmental friendliness of production using biological fillers. For this reason, research on bio-fillers that are potentially applicable in SLA technology is essential [30]. These fillers offer a strong interface with the polymer matrix and also help in filling voids, and thus they have a positive effect on many mechanical properties, especially by transferring stress from the matrix to the reinforcement [31].

The potential limitations of adding opaque fillers to photosensitive resins may be the reduction in photosensitivity and deterioration of mechanical properties through imperfect UV curing of the composite [15,16,32]. It is also reasonable to expect that problems with the preparation of the photosensitive resin and the actual printing process will also arise as the amount of filler and size of filler increases [3,33,34]. Extensive research and innovation are essential in this field given the potential of using different types of fillers in polymer composites. This is a potentially important advantage of SLA 3D-printing technology [5,35,36], which deserves a lot of research in the field of epoxy-based polymer composite materials developed by FDM printing [37]. Another important aspect is the modification of viscosity of the SLA resin by adding different types, sizes and concentrations of fillers (e.g., glass, carbon, nylon, ABS, nanocellulose, woods, etc.) [5,7,8,15,35]. By adding fillers to the SLA resin, the isotropic properties can be changed to anisotropic properties subsequently after the 3D-printing process [2]. For this reason, some researchers have been concerned about modifying the orientation and arrangement of the filaments, e.g., by ultrasound-directed self-assembly (DSA) [2].

## 2. Materials and Methods

### 2.1. Materials

#### 2.1.1. Test Body

**Figure 1:**
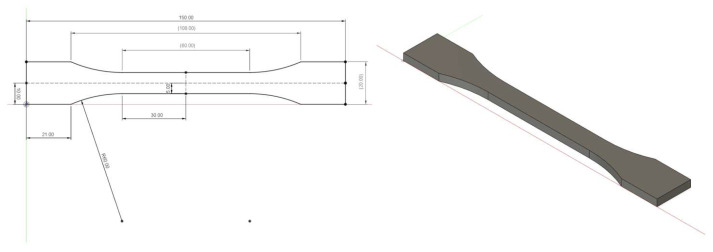
Dimensions and 3D model of the test specimen as per standard, EN-ISO-527-2:2012.

The test specimens were manufactured as per the standard “ČSN EN ISO 527-2:2012 Plastics—Determination of tensile properties—Part 2: Test conditions for moulding and extrusion plastics” ( especially the test specimen 1B). This specimen has an overall length of 150 mm, a width of 10 mm in the tapered part and a uniform thickness of 4 mm along the body. The overall dimensions of the test specimen are given in Figure 1. The Fusion 360 software (product version 2.0.20948, Autodesk, Ltd., San Francisco, CA, USA) was used for creating the 3D model of the test sample. Subsequently, the 3D model was exported to STL format. Additional data are provided in Supplementary Materials.

#### 2.1.2. Resin and Fillers

**Figure 2:**
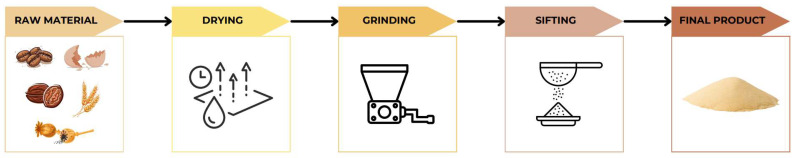
Filler preparation process.

The photosensitive resin for developing the composite samples was chosen from the manufacturer Anycubic. It is a UV resin with colour pigments (sourced from ANYCUBIC Technology Company Limited., Shenzhen, Guangdong Province, China). The composition of this resin is polyurethane acrylate, acrylate monomer and a photo indicator. A transparent colour was preferred to avoid the possible pigmentation and its effect on the mechanical performance. This photosensitive resin is curable by using UV light with a wavelength of 405 nm. Both fibre and particle-type fillers were used in this research. These fillers are obtained as by-products of post-harvest processing of various biological commodities. By-products from post-harvesting processes in Europe and Asia were collected and dried at 105 ± 5 °C for 24 h. Thereafter, the input materials were ground by using multiple stages in an industrial crusher and mill (purchased from company Stránský a Petržík, Pneumatické válce s r.o., Bílá Třemešná, Czech Republic). This device has a hopper capacity of 0.5 kg and input power of 1.1 kW. The materials/fillers that have been ground were sorted by sieve analysis, i.e., by performing the fractionisation method using a Haver EML digital plus machine. The preparation procedure of the different types of fillers is presented in Figure 2.

Eleven diverse types of bio-fillers were used to produce the composite materials through MSLA 3D-printing technology. The filler type, designation and concentration (ratio by weight) are given in Table 1. The concentration of a given filler in the photosensitive resins was determined on weight basis (%). This method was also used by several researchers dealing with the addition of various bio-fillers to photosensitive resins. For this research, the weight % of the bio-fillers was used to simplify the production of composites with expected practical applications. It was also aimed at keeping the printing parameters constant, as given in Table 2. In the case of higher quantities, optimisation of the printing parameters would be necessary, and this would not allow a proper comparison in the case of a larger group of fillers.

Several researchers dealing with the addition of various types of bio-fillers to photosensitive resin using SLA 3D-printing technology used weight % to determine the concentration of a given filler [3,34,38,39,40]. The materials investigated in this research can be categorised as polymer composites with fragmented reinforcement types, e.g., particulates or short fibre-based fillers. These composite materials are applicable in those products where easy processability and relatively good mechanical performance are required [41]. The fillers used in this research have not been chemically treated.

### 2.2. Methods

#### 2.2.1. 3D Printer Device and Parameters of Printing

**Figure 3:**
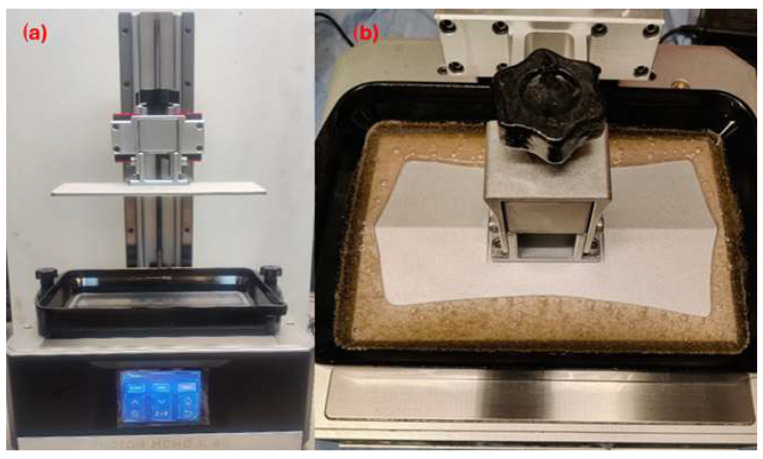
MSLA printer. (**a**) Photon-based Mono × 6 K device, (**b**) composite compound printing.

A printer using MSLA technology was used to produce the test specimens. This is a Photon based Mono × 6K device (developed by ANYCUBIC Tech. Co., Ltd., Shenzhen, Guangdong Province, China). The printer display has a resolution of 5760 × 3600 pixels (as shown in Figure 3). The test specimens were prepared for printing using special software named CHITUBOX (product version 1.9.4., CBD-Tech. Co., Ltd., Shenzhen, Guangdong Province, China). The printing parameters listed in Table 2 were set to produce all the test specimens. The setting of these parameters was based on the recommendations of the resin manufacturer and allows the comparison of unfilled resin with modified resin using bio-fillers.

#### 2.2.2. Scanning Electron Microscopy (SEM)

**Figure 4:**
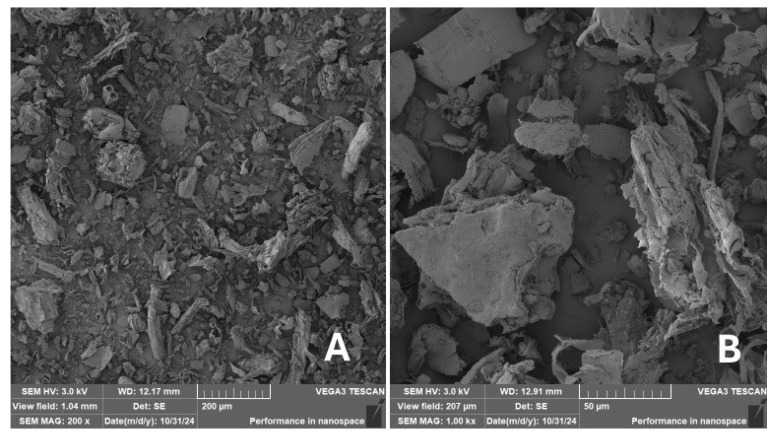
Scanning electron microscopy of the CT-type filler in the polymer composite material: (**A**) microparticles CT (200×), (**B**) higher magnification of the microparticles CT (1.00 k×).

**Figure 5:**
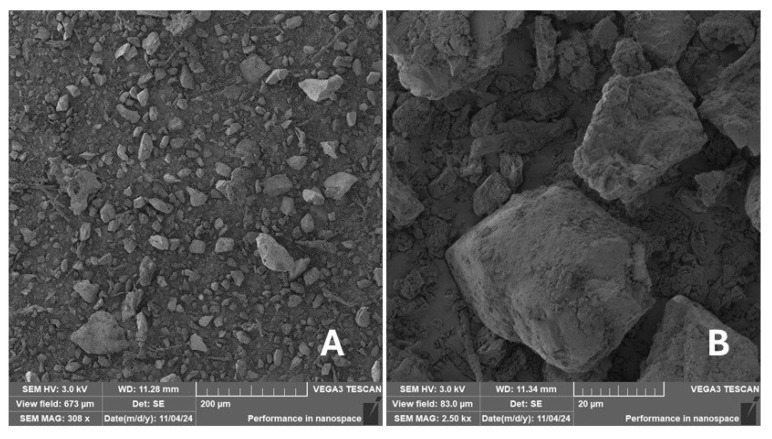
Scanning electron microscopy of the ES-type filler in the polymer composite material: (**A**) microparticles ES (308×), (**B**) higher magnification of the microparticles ES (2.50 k×).

**Figure 6:**
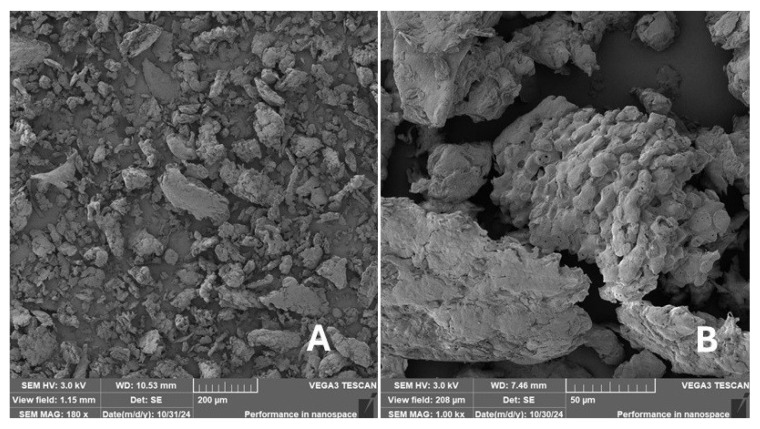
Scanning electron microscopy of the WT-type filler in the polymer composite material: (**A**) microparticles WT (180×), (**B**) higher magnification of the microparticles WT (1.00 k×).

**Figure 7:**
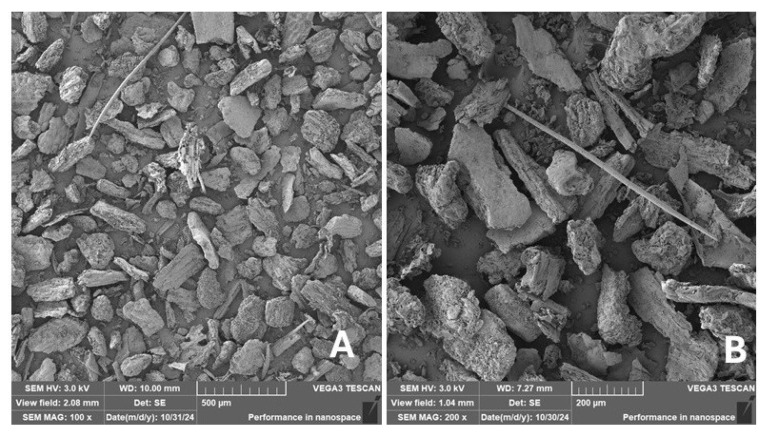
Scanning electron microscopy of the PP-type filler in the polymer composite material: (**A**) microparticles PP (100×), (**B**) higher magnification of the microparticles PP (200×).

**Figure 8:**
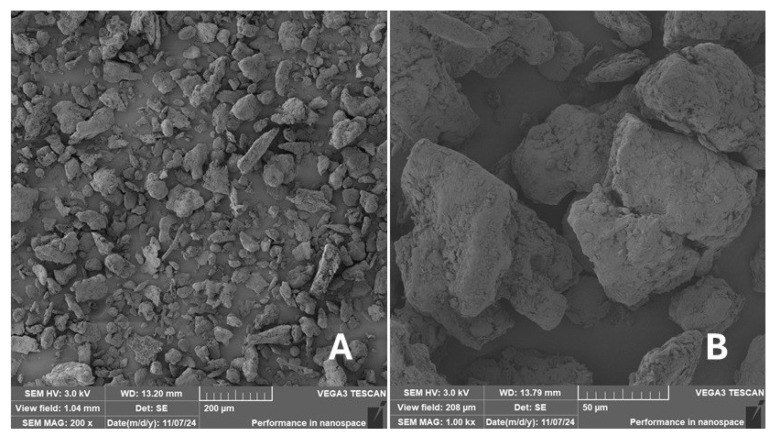
Scanning electron microscopy of the PA-type filler in the polymer composite material: (**A**) microparticles PA (200×), (**B**) higher magnification of the microparticles PA (1.00 k×).

**Figure 9:**
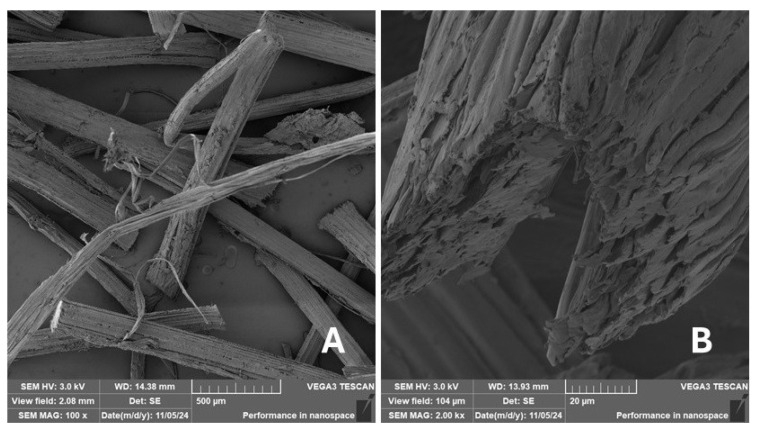
Scanning electron microscopy of the BF-type filler in the polymer composite material: (**A**) microfibres BF (100×), (**B**) higher magnification of the microfibres BF (2.00 k×).

**Figure 10:**
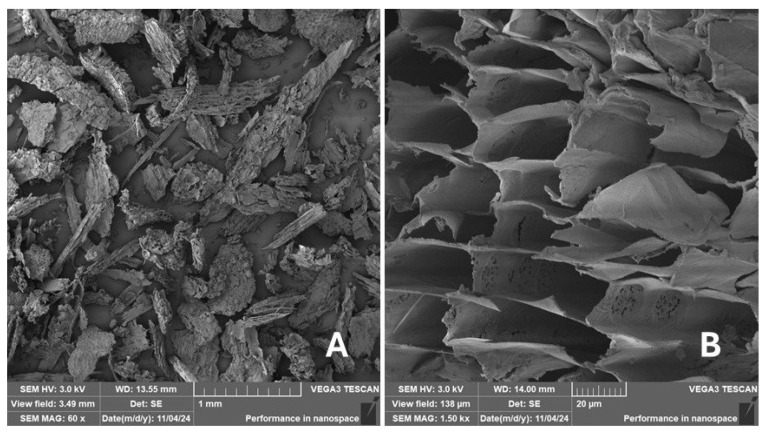
Scanning electron microscopy of the SB-type filler in the polymer composite material: (**A**) microparticles SB (60×), (**B**) higher magnification of the microparticles SB (1.50 k×).

**Figure 11:**
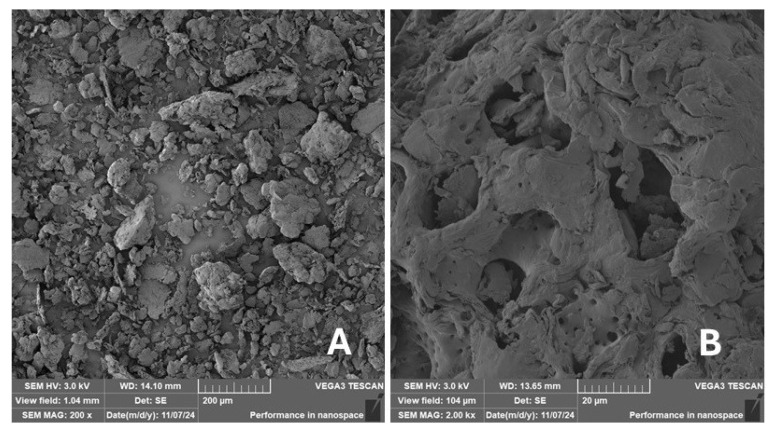
Scanning electron microscopy of the WN-type filler in the polymer composite material: (**A**) microparticles WN (200×), (**B**) higher magnification of the microparticles WN (2.00 k×).

**Figure 12:**
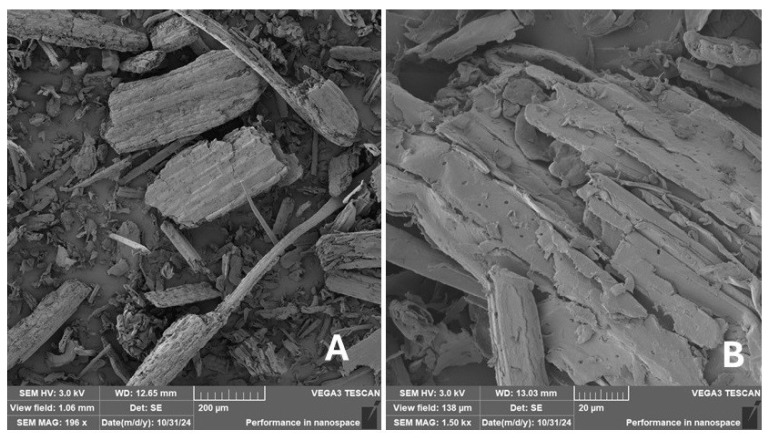
Scanning electron microscopy of the MS-type filler in the polymer composite material: (**A**) microparticles MS (196 ×), (**B**) higher magnification of the microparticles MS (1.50 k×).

**Figure 13:**
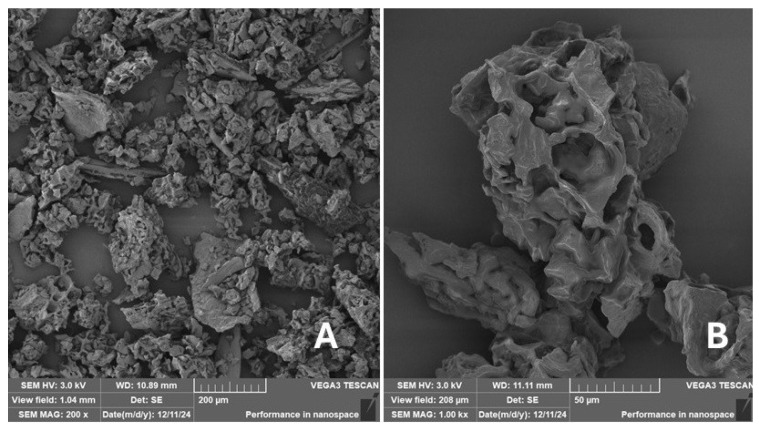
Scanning electron microscopy of the CE-type filler in the polymer composite material: (**A**) microparticles MS (200×), (**B**) higher magnification of the microparticles MS (1.00 k×).

**Figure 14:**
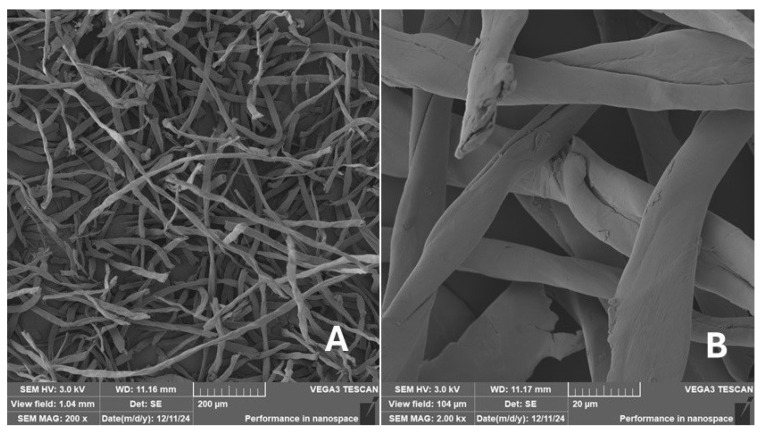
Scanning electron microscopy of the CF-type filler in the polymer composite material: (**A**) microfibres CF (200×), (**B**) higher magnification of the microfibres CF (2.00 k×).

Scanning electron microscopy (SEM) images of the fibrous and particulate bio-fillers used can be seen in Figure 4, Figure 5, Figure 6, Figure 7, Figure 8, Figure 9, Figure 10, Figure 11, Figure 12, Figure 13 and Figure 14. The shapes and varying textures of the surfaces can be seen in Figure 4, Figure 5, Figure 6, Figure 7, Figure 8, Figure 9, Figure 10, Figure 11, Figure 12, Figure 13 and Figure 14. The bio-filler BF (Figure 9), SB (Figure 10) and WN (Figure 11) all exhibited porous internal textures.

The bio-fillers were examined using an SEM device named TESCAN-VEGA-3-XMU (manufactured by TESCAN ORSAY HOLDING a.s., Brno, Czech Republic) having an accelerating voltage of 3 kV. The presented SEM images (Figure 4, Figure 5, Figure 6, Figure 7, Figure 8, Figure 9, Figure 10, Figure 11, Figure 12, Figure 13 and Figure 14) and different magnifications for individual fillers were selected based on the particle size of the different types of fillers. For some types of fillers, the sizes were too large (or too small), so it was not possible to use larger magnifications. It would not be possible to examine the nature of the particles with higher magnification. For this reason, it was not possible to use the same magnification for all fillers. Our effort was to present the overall picture and view of the particles in the best possible way.

The size of individual particle and fibre was obtained by an optical method using the scanning electron microscopy images assisted by Gwyddion software from the same supplier. The results of this analysis are presented in Table 3. The SEM images and Table 3 show the different sizes of the fillers used. The results of image analysis of the fillers are summarised in Table 3. A filler based on the waste from banana tree post-harvest processing (labelled BF) showed an aspect ratio of 11.41. The cotton flakes also showed an aspect ratio of 22.84. This is a short-fibre or filler suitable for the reinforcement of a composite system. Such types of anisotropic fillers show similar behaviour as particulate fillers due to having a similar aspect ratio [42]. The short fibres are shorter than their critical length, which was in the order of 100 times their thickness. The aspect ratio in this case was less than 100 [4].

#### 2.2.3. Preparation Process of the Composite Samples

**Figure 15:**
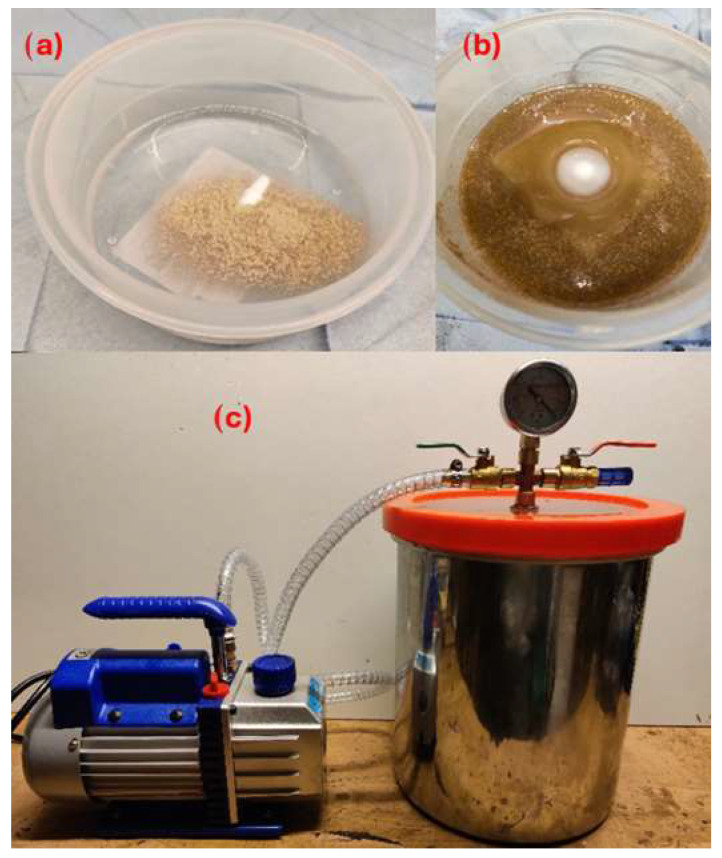
Photosensitive resin and filler mixture (**a**), mixing the resin and fillers using a magnetic stirrer (**b**), vacuum pump (**c**).

The preparation process began by weighing the desired amount of filler and photosensitive resin, which were then mixed into one container (see Figure 15b). The fillers were mixed with the photosensitive resin using a magnetic stirrer, where a magnetic capsule was rotated inside the mixture for 10 min (see Figure 15a), ensuring that the filler is evenly dispersed in the photosensitive resin and avoiding the formation of any clumps of fillers. This part of the preparation process has a significant impact on the storage of particle-filled photosensitive resins and prevents sedimentation of the fillers. If sedimentation issues were not addressed, there would be uneven dispersion of the micro-fillers and there would be a greater number of fillers in the first layers of the test specimens. After 10 mins, large amounts of air bubbling from the mixture could be observed. A vacuum pump was used to remove the air bubbles that would negatively impact the mechanical properties of the fabricated composite samples (see Figure 15c).

**Figure 16:**
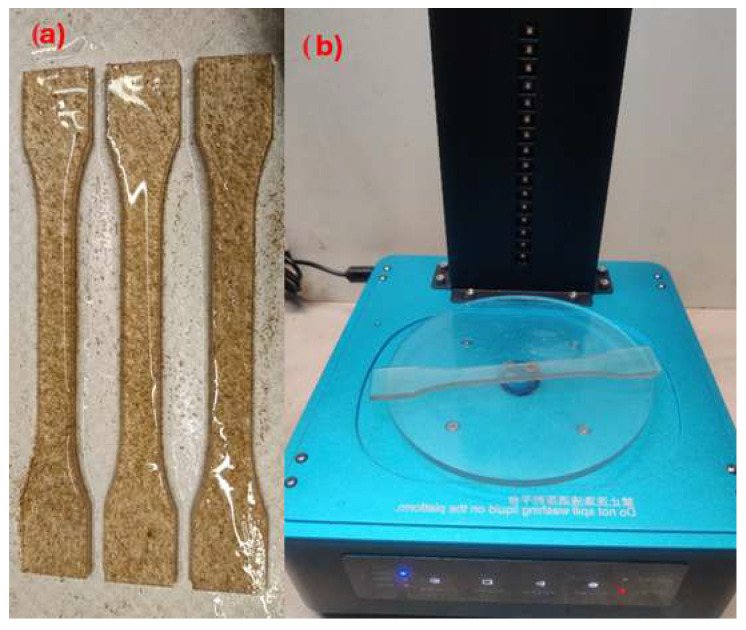
Example of printing a test specimen on the printing platform (**a**), additional curing (**b**).

The filler–resin mixture was then used to produce the composite samples using a MSLA 3D printer (see Figure 16a). The test specimens were then removed from the 3D printer platform, and the excess isopropanol resin was cleaned (recommended by: INCHEMA s.r.o., Prague, Czech Republic). Subsequently, the cleaned specimens were additionally cured in a UV chamber (Figure 16b). Curing was performed by additionally exposing the samples in the UV chamber on a rotating platform exposed to UV light of 405 nm wavelength for 2 mins. Rotation of the sample in the chamber results in uniform curing.

**Figure 17:**
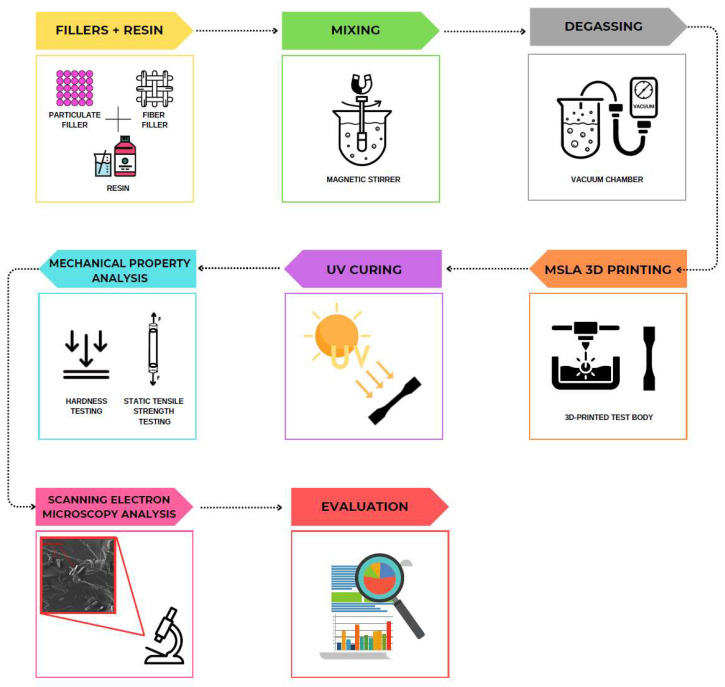
Overall process of manufacturing the composite material using MSLA 3D printing.

The overall process involving the preparation of the mixture and the manufacture of the test specimens is demonstrated in Figure 17. After the production of the test specimens, the mechanical properties were evaluated; see Section 2.2.4 and Section 3.

#### 2.2.4. Testing the Mechanical Properties

The 3D-printed specimens created by the additive SLA 3D-printing technology were evaluated for static tensile loading on the LAB-Test device, model 5.50 ST test rig (developed by LABORTECH s.r.o., Opava, Czech Republic). The device was equipped with the AST KAF system having a measuring unit of 50 kN (LABORTECH s.r.o., Opava, Czech Republic) and the associated software for evaluation (Test&Motion, product version 4.5.0.15, from LABORTECH s.r.o, Opava, Czech Republic). The standards ČSN EN ISO 527-1, Plastics—Determination of tensile properties—Part 1: General principles and hardness measurements using a DuraJet G5 hardness tester (developed by Struers GmbH, Roztoky u Prahy, Czech Republic) and ČSN EN ISO 2039-1 (64 0619) Plastics—Determination of hardness—Part 1: Ball indentation method were used. Hardened stainless steel balls having a diameter of 5 mm were used. The test conditions included an initial load of 9.8 N and test load of 132 N for testing the hardness of the samples.

Differently sized test specimens were used for each type of material produced by SLA 3D printing to investigate the mechanical properties. For comparison purposes, a photosensitive SLA resin (i.e., a UV resin having colour (supplied by ANYCUBIC Tech. Co., Ltd., Shenzhen, Guangdong Province, China) was used. It was available in a coloured transparent state and without any fillers.

#### 2.2.5. Scanning Electron Microscopy (SEM) Analysis

The specimens printed by SLA 3D-printing technology were tested for tensile performance and were fractured. Such samples were examined by using the device TESCAN VEGA 3 XMU (TESCAN ORSAY HOLDING a.s., Brno, Czech Republic). The device has an accelerating voltage of 3 kV. For SEM analysis of fracture surfaces of composite materials, the broken specimens after static tensile testing were used. An approximately 15 mm long part of the test specimen along with the fractured surface was cut using a saw with precision metallography. The emphasis was on avoiding damage or contamination of the fractured surface. The test samples selected by this method were then glued to aluminium stubs (sample holders—aluminium stub supplied by TESCAN ORSAY HOLDING as, Brno, Czech Republic) using double-sided adhesive carbon discs and properly labelled so as to avoid any confusion among the samples. Subsequently, the samples were placed in a Quorum 150R ES-sputtering deposition machine (Quorum Technologies Ltd., UK), and a 20 nm layer of Au was sputtered onto the fractured surface of the samples to create a conductive Au layer for subsequent observations using SEM, since both the photosensitive resin and the fillers were non-conductive materials.

## 3. Results and Discussion

**Figure 18:**
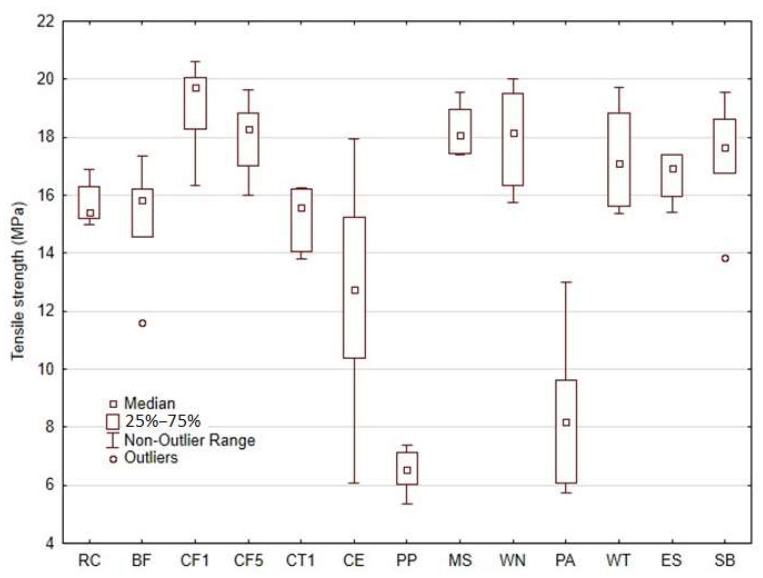
Tensile strength of photosensitive resin RC and composite materials using different bio-fillers.

The tensile test results of the 3D-printed composite materials using a matrix of photosensitive resin and various types of fillers based on natural secondary raw materials are shown in Figure 18.

The results of the tensile tests clearly indicate the positive effects of most types of natural particulates and fibre-based fillers in the composite. From Figure 18, it can be seen that the most significant increase in the tensile strength (by up to approximately 22%) was achieved by using the microparticle filler labelled CF1 (cotton flakes). For the other types of fillers labelled CF5, MS, WN, WT, ES and SB, the increase in tensile strength ranged from 6 to 16%. There was no significant increase in the standard deviation value (SD) for the filler type RC. This indicates a homogeneous distribution of fillers in the composite samples.

Further, the variance of the results obtained ranged from about 4 to 13%. The photosensitive resin without any filler, labelled as RC, showed a tensile strength of 15.70 ± 0.73 MPa (with 5% variance).

Therefore, the primary assumption of a significant environmental effect through the greater use of natural materials in composite manufacturing was established [30]. The possibility of improving mechanical properties through the use of different types of bio-fillers in the composites was confirmed [31]. The assumptions of there being a significant limitation of this 3D-printing technology and the deterioration of the UV-cured photosensitive resins leading to a deterioration of mechanical properties were not confirmed [16,32,33]. The use of biological filler type CE (microparticles of coffee grounds) and PA (microparticles of palm) resulted in a significant reduction in the tensile strength but also increased the variance of the results. For the microparticle type CE, there was a 20.28% decrease in tensile strength, (i.e., 12.52 ± 4.13 MPa). The microparticle type PA resulted in a significant reduction in tensile strength of up to 46.01%, (i.e., 8.48 ± 2.71 MPa). The CE and PA-type fillers showed the maximum variance in results (approximately 32%).

The increased variance of the results can be attributed to the unavoidable variations in the preparation of the materials, the printing process itself and the significant change in the viscosity of the prepared matrix, which has been pointed out by several researchers in this area [5,7,8]. The use of PP fillers (microparticles from poppy) resulted in a significant reduction in the tensile strength. In case of microparticle type PA, there was a decrease in strength up to 58.61%, (i.e., 6.50 ± 0.78 MPa), which was also characterised by a variance of about 12%.

From the tensile strength results, it is clear that affordable SLA 3D-printed materials cured at 405 nm UV light can be modified with selected types of natural fillers and can achieve improvement in the mechanical properties.

The use of filler materials is one of the ways to eliminate the limitations of SLA 3D-printing technology, which otherwise results in reduced mechanical properties compared to FDM printing [3].

**Figure 19:**
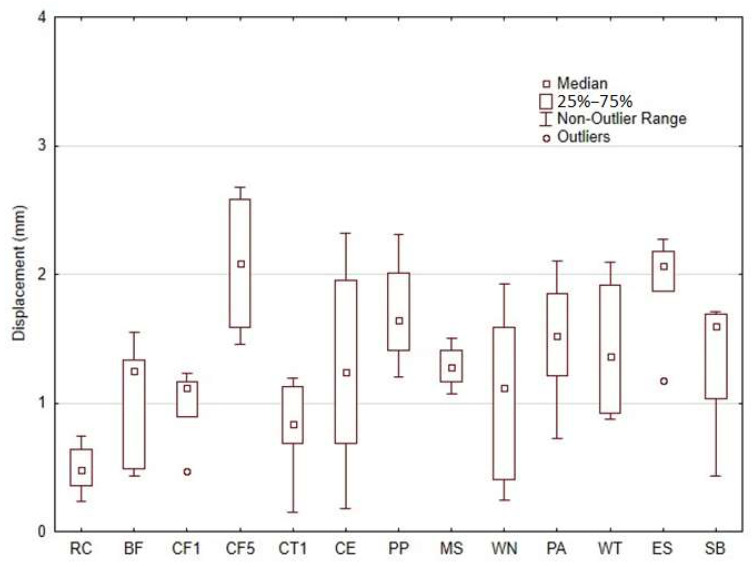
Displacement of photosensitive resin RC and composite materials using different bio-fillers.

The use of bio-fillers is considered a highly encouraging option for additive 3D-printing technologies because of the wide range of properties and their easy availability from various sustainable resources [24]. High tensile strength is often an essential and non-negotiable requirement in real industrial applications, especially when dealing with structural components, where the durability, reliability and safety are of utmost importance [25]. Figure 19 shows the displacement at break for various samples. It can be observed that the 3D-printed material with RC-type filler exhibited a breaking elongation value of 0.49 ± 0.18 mm. The other materials with the addition of different bio-fillers showed an increase in displacement in the range of 63% to 323%. Thus, it is evident from the obtained results that the inclusion of natural particulate and fibrous fillers in the photosensitive resin has modified the material printed by AM MSLA technology. It resulted in a significant increase in the breaking elongation. No significant difference in the change in displacement between the microparticulate and fibre fillers was demonstrated. The impact of varying concentrations of cotton flakes (CF) filler was also examined. The results indicated a positive trend in the enhancement of mechanical properties as the filler concentration increased.

**Figure 20:**
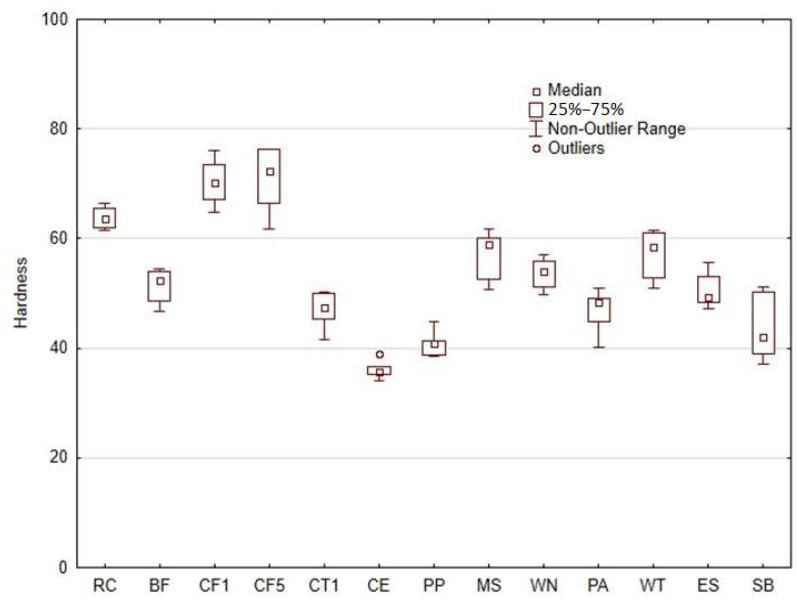
Hardness of photosensitive resin RC and composite materials using different bio-fillers.

Figure 20 illustrates the hardness of samples. It shows that the inclusion of the bio-fillers does not have a significant effect on the hardness. As compared to the pure photosensitive resin printed by AM MSLA technology, which was 63.87 ± 1.94, there was a slight increase or decrease in the hardness. Only the cotton flakes-based fillers resulted in an increase in hardness of about 10%. The other types of fillers showed a decrease in hardness ranging from 16 to 43%. It is therefore clear from the results that the addition of bio-fillers tends to reduce the resulting hardness. There was a significant increase in hardness expected when using bio-fillers, as is usually the case when using a wear-resistant filler added to polymers, e.g., coconut shell microparticles, Al_2_O_3_, SiC, etc. [43,44]. The presence of bio-fillers used in additive 3D-printing technology improves their environmental impact [34,45]. Furthermore, the relatively lower density of the matrix (resin+filler) is an important advantage. The inclusion of natural fillers has also been noted as a successful industrial method for improving the performance of various photo-cures.

**Figure 21:**
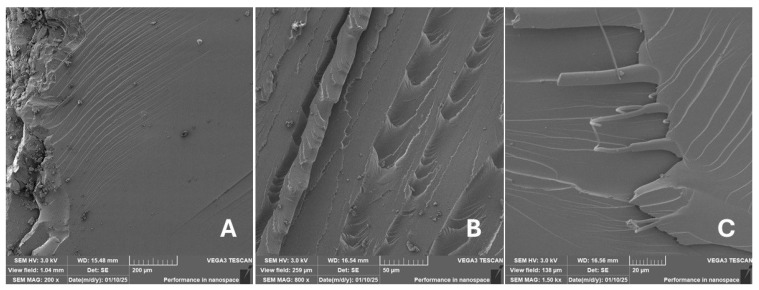
Scanning electron microscopy of the fractured surface after static tensile test of sample RC: (**A**) 200×, (**B**) 800×, (**C**) 1.50 k×.

**Figure 22:**
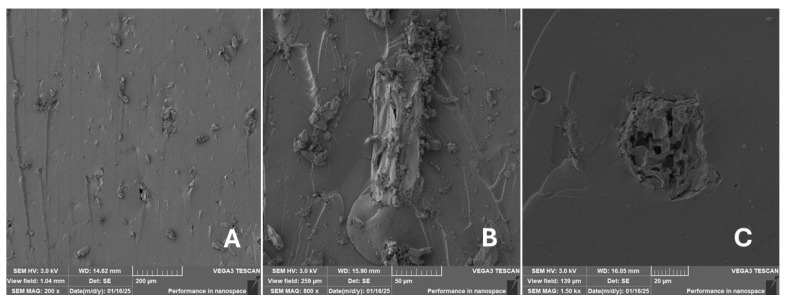
Scanning electron microscopy of the fractured surface after static tensile test of sample CT: (**A**) 200×, (**B**) 800×, (**C**) 1.50 k×.

**Figure 23:**
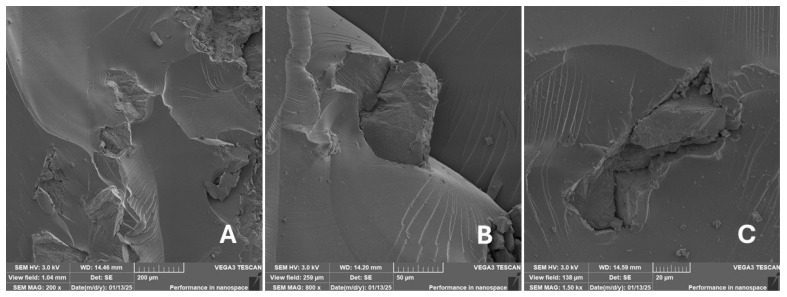
Scanning electron microscopy of the fractured surface after static tensile test of sample ES: (**A**) 200×, (**B**) 800×, (**C**) 1.50 k×.

**Figure 24:**
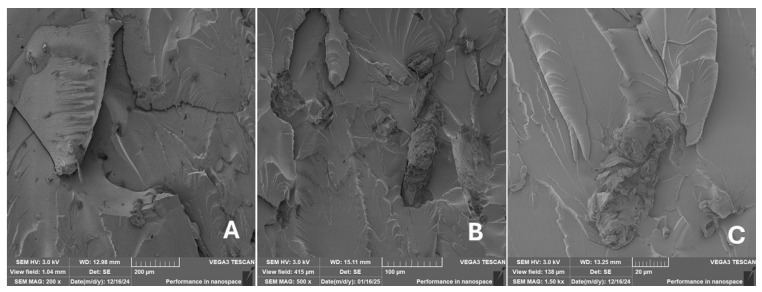
Scanning electron microscopy of the fractured surface after static tensile test of sample WT: (**A**) 200×, (**B**) 800×, (**C**) 1.50 k×.

**Figure 25:**
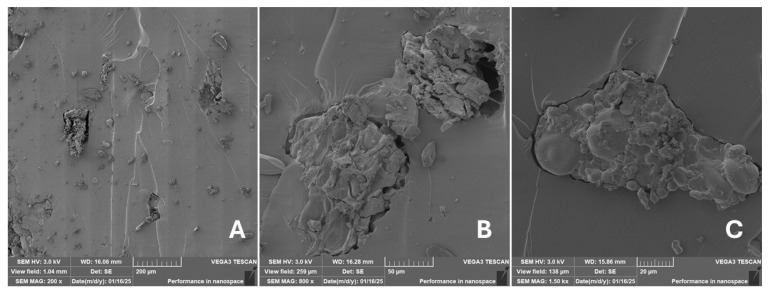
Scanning electron microscopy of the fractured surface after static tensile test of sample PP: (**A**) 200×, (**B**) 800×, (**C**) 1.50 k×.

**Figure 26:**
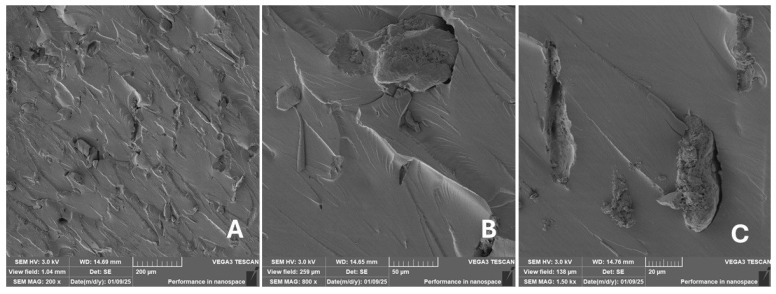
Scanning electron microscopy of the fractured surface after static tensile test of sample PA: (**A**) 200×, (**B**) 800×, (**C**) 1.50 k×.

**Figure 27:**
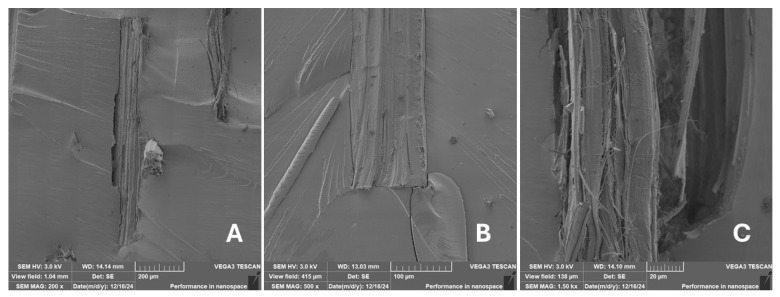
Scanning electron microscopy of the fractured surface after static tensile test of sample BF: (**A**) 200×, (**B**) 800×, (**C**) 1.50 k×.

**Figure 28:**
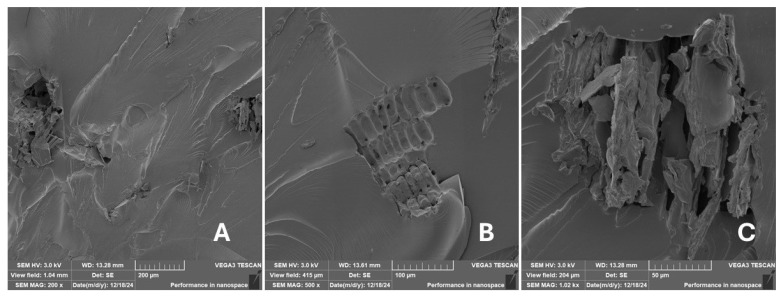
Scanning electron microscopy of the fractured surface after static tensile test of sample SB: (**A**) 200×, (**B**) 800×, (**C**) 1.50 k×.

**Figure 29:**
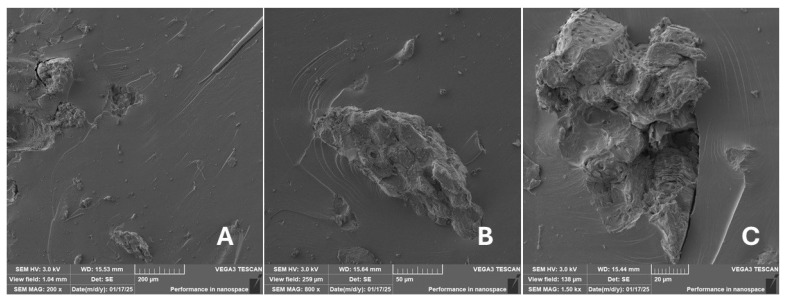
Scanning electron microscopy of the fractured surface after static tensile test of sample WN: (**A**) 200×, (**B**) 800×, (**C**) 1.50 k×.

**Figure 30:**
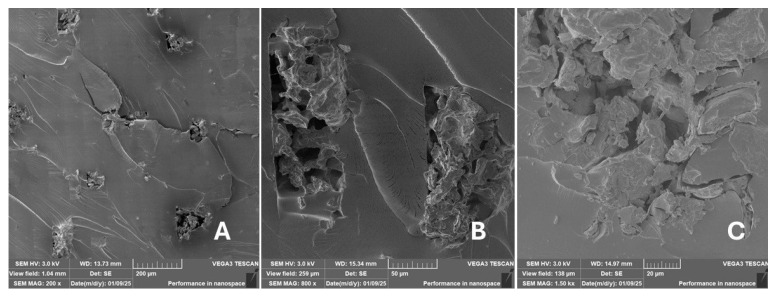
Scanning electron microscopy of the fractured surface after static tensile test of sample CE: (**A**) 200×, (**B**) 800×, (**C**) 1.50 k×.

**Figure 31:**
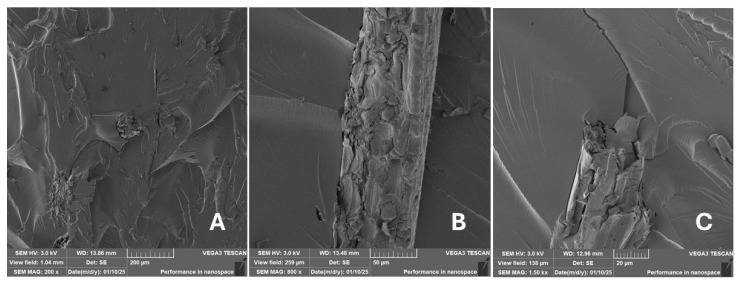
Scanning electron microscopy of the fractured surface after static tensile test of sample MS: (**A**) 200×, (**B**) 800×, (**C**) 1.50 k×.

**Figure 32:**
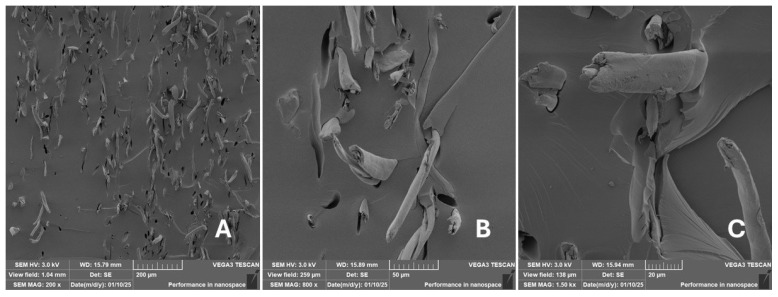
Scanning electron microscopy of the fractured surface after static tensile test of sample CF: (**A**) 200×, (**B**) 800×, (**C**) 1.50 k×.

Incorporating natural fibres has also been noted as a successful strategy to improve the performance of various photo-cured resins to develop composites by using SLA 3D-printing technology [34,38]. The results of the measurements clearly demonstrate a positive trend, especially by increased tensile strength values. Different 3D-printing methods and modifications to the base printing materials involve risks that must be eliminated. Research in the characterisation of the finished product contributes to the overall understanding of the performance of polymeric composites prepared through the technology of additive manufacturing, which allows for optimisation and quality improvement [9]. The quality and mechanical performance of polymeric materials developed by SLA additive manufacturing technology are crucial in the evaluation of these materials [46]. In particular, scanning electron microscopy (SEM) is essential in this research, as it allows for the acquisition of high-resolution images, which facilitates a comprehensive investigation of the various physical properties of the composite materials [9]. The morphology of fractured surfaces and composite materials based on natural secondary by-products from agricultural crop processing produced by AM SLA 3D-printing technology can be seen in Figure 21, Figure 22, Figure 23, Figure 24, Figure 25, Figure 26, Figure 27, Figure 28, Figure 29, Figure 30, Figure 31 and Figure 32. In Figure 21, Figure 22, Figure 23, Figure 24, Figure 25, Figure 26, Figure 27, Figure 28, Figure 29, Figure 30, Figure 31 and Figure 32, label A represents the fractured surface at low magnification. The figures labelled B and C (in Figure 21, Figure 22, Figure 23, Figure 24, Figure 25, Figure 26, Figure 27, Figure 28, Figure 29, Figure 30, Figure 31 and Figure 32) then provide a higher magnified view of the fractured material surface, the geometric shape of the bio-filler and its interaction with the photosensitive resin. From the obtained results of the mechanical performance, it is evident that the fillers investigated in this work significantly affect the mechanical properties. The observed results of the scanning electron microscopy indicate how the individual fillers affected the integrity of the tested composite material.

The photosensitive RC resins exhibit a characteristic brittle fracture in static tensile tests, which can be seen in Figure 21. Similar behaviour is seen in two-component epoxy resins [43]. A higher magnification image of the fractured sample surface can be visible in Figure 21B,C.

The fractured surfaces of all the composite samples can be characterised by two areas; namely fracture initiation and fracture development, which can be seen in Figure 21A. The fracture development area is characterised by surface fragmentation in Figure 21B,C. The majority of the fractured surface of sample RC shows a smooth profile without significant fracture relief (Figure 21A). The addition of filler interrupted the development of surface fragmentation. Nevertheless, other samples prepared with bio-fillers show areas of distinct relief in the fracture surface.

The presence of various types of natural fillers, based on microparticles and random fibres, are clearly visible on the fractured surface of the tensile tested samples shown in Figure 22, Figure 23, Figure 24, Figure 25, Figure 26, Figure 27, Figure 28, Figure 29, Figure 30, Figure 31 and Figure 32. It is evident that the fillers are embedded in the matrix with very good interaction with the resin, as visible in Figure 24C, Figure 27B, Figure 30C, etc. But on the other hand, there are certain fillers that show relatively poor interaction with the matrix; a few examples are shown in Figure 25B,C and Figure 26B,C. Some SEM images also show the destruction of individual fillers; i.e., their cohesive strength was lower than the adhesive strength with the matrix, e.g., Figure 27C and Figure 32C. It is also visible on the fractured surface in Figure 32 that the fibres are pulled out from the polymer matrix. Similar results from SEM analysis were also obtained by the investigation of keratin-rich fibres from rabbit hair in additive SLA printing, where the voids on the fractured surface were caused by the pulled-out fibres [34]. Such a negative trend can be eliminated by modifying the fibre surfaces [11,34,47,48,49]. The resulting random fibre arrangement might have a critical role in the process of transferring stress from the matrix to the fibre phase [50]. Nevertheless, the use of short, non-oriented fibres can be viewed with several advantages. The reason is that at relatively lower concentrations, they have the potential for the effective prevention of crack propagation [51]. The arrangement of cotton flakes (CF) and banana fibre (BF) microfibres can be seen in Figure 27 and Figure 32.

Cotton fibres are irregular in their shape and length and have a smooth surface. The fibres are significantly flexible and offer a better adhesion with the polymer matrix. However, when the fibres are pulled out, there are micro-voids visible on the fractured polymer material surface. Neither the matrix, i.e., the photosensitive RC resin, nor the composite materials exhibited porosity on the fracture surface during SLA 3D-printing technology. A very low amount of porosity was found only in samples where oil palm (PA) microparticles were used as fillers (shown in Figure 26A). Yadollahi and Shamsaei point out the porosity in additive technology and state that it may be due to either inappropriate manufacturing parameters or filler addition [52]. They pointed out that porosity can result in a reduction in mechanical properties, which has been demonstrated in the literature [52]. PA filler-based samples with oil palm microparticles resulted in a significant reduction in the tensile strength.

It was evident from the obtained results that the compatibility of various types of biobased particulates and fibre fillers with the photosensitive resin is good at the interface and has the potential for broader applications. The overall porosity of the composite samples was relatively low. Elevated values were found by using an oil palm (PA) microparticle-based filler. Investigations of the coconut shell-based micro-fillers indicated the significant presence of oil [53]. A low value of porosity (8%) was reported for samples using fillers in SLA 3D-printing technology [54]. Also, the microparticle fillers based on coffee grounds (CE) and oil palm (PA) showed a significant variance in tensile strength results because of their inherent porosity in the fillers, which might have led to reduced cohesion as seen in Figure 26 and Figure 30B,C. In the evaluation of fractured surfaces using fillers, it is common to observe the filler being pulled out of the matrix or being destroyed [34]. The interface between the matrix and both particulate and fibrous fillers is very important for the overall integrity of the composite materials [51,55].

The limitations of this 3D-printing technology to produce composites using photosensitive resins with fillers should also be considered seriously. One of the limitations of this technology using fillers is the storage of such resins, particularly due to risks of filler sedimentation. Because of sedimentation, it is necessary to mix the matrix thoroughly with the filler before 3D printing using rotary stirrers, as explained in Section 2.2.3.

## 4. Conclusions

The current research investigated the feasibility of incorporating eleven different bio-fillers into a commercially available photosensitive resin designed for additive SLA 3D-printing technology. Additive manufacturing technology with the inclusion of natural origin bio-filler materials represents a significant potential for new age composite materials. A key benefit of the proposed approach is an alternative use of otherwise waste biomaterials through appropriate recycling, which has a significant impact on the circular economy and waste management. Using even a small amount (e.g., 3%) of natural filler can reduce the overall plastic content of the final composite product. At the same time, there are industrial requirements to incorporate natural fillers into the mass production of composite components even in very small quantities. The materials printed by using SLA technology exhibited enhanced mechanical properties with all the types of bio-fillers, revealing varying degrees of improvement in tensile strength. This research has demonstrated the possibility of the effective modification of photosensitive resin especially designed for AM SLA 3D printing.

It was observed from the results obtained that the inclusion of natural bio-fillers in photosensitive resins has a limited effect on the enhancement of hardness. Rather, most of the natural fillers used resulted in a slight reduction in hardness. The only exception was in case of cotton flakes (CF), which caused a slight increase in hardness. However, in terms of mechanical properties, a positive effect was observed in improving the tensile strength.

Significant increases in tensile strength (21.8%) were achieved for composites filled with cotton flakes (CF), while the increases were smaller for the other cases: 16.3% for miscanthus (MS), 14.5% for walnut (WN), 10.5% for spruce tree (SB), 10.1% for wheat (WT), and 6.2% for eggshell (ES). On the other hand, oil palm (PA) fillers decreased the tensile strength by up to 46.1%, poppy (PP) fillers by up to 58.6% and coffee grounds (CE) fillers by 20.2%.

The mechanical properties were affected differently based on the nature and variety of the bio-fillers used and their interfacial bond with the matrix. SEM analysis revealed a different quality of bonding between the filler and matrix. The SEM analysis revealed very good adhesion for some types of fillers but also porosity and weakened cohesion for some others, especially for the fillers based on oil palm microparticles (labelled PA). Nevertheless, natural bio-fillers offer significant potential for improving certain properties in a 3D-printed composite material and contribute to the positive environmental impact. It is necessary to optimise their incorporation into the 3D-printed composite material, which has been demonstrated by the current research.

The mechanical tests indicated a generally positive trend: that the addition of bio-fillers increases tensile strength and helps achieve better mechanical properties through effective filler–matrix interaction. One of the most important findings is the fact that modifying reactoplasts with bio-fillers yields an increase in tensile strength in the order of ten percent. It was further demonstrated which types of fillers are not suitable, e.g., coffee grounds (CE), poppy process waste and coconut husk. It has also been shown that there is a slight decrease in hardness in the developed composite materials, which was not expected. This research highlights the potential of various types of bio-fillers, paving the way for further investigations. Future research could explore different filler concentrations, surface modifications, degradation processes, and other factors that are essential for a range of practical applications using additive stereolithography (SLA) 3D-printing technology. The fillers based on microfibres like cotton flakes CF demonstrated having a significant impact on the mechanical properties. These biobased fillers have significant potential for further research activities and value addition to the 3D-printed composite materials.

## Figures and Tables

**Table 1:** Overview of used biological fillers and their weight% in the matrix (photosensitive resin).

Matrix	Type of Filler	Proportion of Filler to Matrix [%wt.]	Specimen ID
Clear UV resin	-	-	RC
Clear UV resin	Cotton	1.4	CT1
Clear UV resin	Eggshells	3.0	ES
Clear UV resin	Wheat	3.0	WT
Clear UV resin	Poppy	3.0	PP
Clear UV resin	Palm	3.0	PA
Clear UV resin	Banana	2.0	BF
Clear UV resin	Spruce tree	1.3	SB
Clear UV resin	Walnut	1.3	WN
Clear UV resin	Miscanthus	1.3	MS
Clear UV resin	Coffee grounds	1.3	CE
Clear UV resin	Cotton flakes	5.0	CF5
Clear UV resin	Cotton flakes	1.0	CF1

**Table 2:** The parameters for MSLA printing.

Printing Parameters	Value
Height of layer	0.05 mm
Time of exposure	2 s
Antialiasing	1
Distance of lifting	8 mm
Speed of lifting	2 mm/s
Time of bottom exposure	23 s
Count of bottom layer	6
Distance of retract	8 mm
Speed of restarting	3 mm/s

**Table 3:** Parameters of the fillers used for preparation of composite material using MSLA 3D-printing technology.

Filler Designation	Filler Type	Arithmetic Mean (µm)	Median (µm)	Mode (µm)
CT—Cotton	Particle	25.0	18.0	15.0
ES—Eggshells	Particle	254.0	8.4	7.8
WT—Wheat	Particle	45.4	43.0	32.0
PP—Poppy	Particle	106.9	87.0	43.0
PA—Palm	Particle	32.1	28.0	23.0
BF-L—Banana	Short fibres	1449.6	1535.0	1807.0
BF-D—Banana	Particle	127.0	121.0	90.0
SB—Spruce tree	Particle	254.0	199.0	71.0
WN—Walnut	Particle	33.8	30.0	30.0
MS—Miscanthus	Particle	128.0	79.5	42.0
CE—Coffee grounds	Particle	59.2	52.0	52.0
CF-L—Cotton flakes	Short fibres	342.2	318.5	307.0
CF-D—Cotton flakes	Short fibres	14.9	15.0	19.0

## Data Availability

The original contributions presented in this study are included in the article/Supplementary Materials. Further inquiries can be directed to the corresponding author(s).

## Supplementary Materials

The following supporting information can be downloaded at: https://www.mdpi.com/article/10.3390/ma18122699/s1, Additional data are provided in Supplementary Materials.
